# Coronary Atherosclerotic Disease and Cancer: Risk Factors and Interrelation

**DOI:** 10.3389/fcvm.2022.821267

**Published:** 2022-04-07

**Authors:** Jinjing Li, Jieqiong Zhao, Yonghong Lei, Yan Chen, Miaomiao Cheng, Xiaoqing Wei, Jing Liu, Pengyun Liu, Ruirui Chen, Xiaoqing Yin, Lei Shang, Xue Li

**Affiliations:** ^1^Department of Cardiology, Tangdu Hospital, The Fourth Military Medical University, Xi’an, China; ^2^Department of Plastic Surgery, Chinese PLA General Hospital, Beijing, China; ^3^Department of Cardiology, People’s Hospital of Taishan, Taishan, China; ^4^Department of Health Statistics, School of Public Health, The Fourth Military Medical University, Xi’an, China

**Keywords:** coronary atherosclerosis, cancer, risk factors, prediction model, ALT

## Abstract

**Background:**

In our clinical work, we found that cancer patients were susceptible to coronary atherosclerotic heart disease (CAD). However, less is known about the relationship between CAD and cancer. The present study aimed to identify the risk factors for CAD and cancer, as well as the relationship between CAD and cancer.

**Methods:**

In this retrospective study, 1600 patients between January 2012 and June 2019 were enrolled and divided into groups according to whether they had CAD or cancer. Single-factor and multivariate analysis methods were applied to examine the risk factors for CAD and cancer.

**Results:**

(1) Cancer prevalence was significantly higher in patients with CAD than in patients without CAD (47.2 vs. 20.9%). The prevalence of CAD in cancer and non-cancer patients was 78.9 and 52.4%, respectively. (2) Multivariable logistic regression showed that patients with cancer had a higher risk of developing CAD than non-cancer patients (OR: 2.024, 95% CI: 1.475 to 2.778, *p* < 0.001). Respiratory (OR: 1.981, 95% CI: 1.236–3.175, *p* = 0.005), digestive (OR: 1.899, 95% CI: 1.177–3.064, *p* = 0.009) and urogenital (OR: 3.595, 95% CI: 1.696–7.620, *p* = 0.001) cancers were significantly associated with a higher risk of CAD compared with no cancer. (3) Patients with CAD also had a higher risk of developing cancer than non-CAD patients (OR = 2.157, 95% CI: 1.603 to 2.902, *p* < 0.001). Patients in the Alanine aminotransferase (ALT) level ≥ 40 U/L group had a lower risk of cancer than patients in the ALT level < 20 U/L group (OR: 0.490, 95% CI: 0.333–0.722, *p* < 0.001). (4) An integrated variable (Y = 0.205 × 10^–1^ age − 0.595 × 10^–2^ HGB − 0.116 × 10^–1^ ALT + 0.135 FIB) was identified for monitoring the occurrence of cancer among CAD patients, with an AUC of 0.720 and clinical sensitivity/specificity of 0.617/0.711.

**Conclusion:**

(1) We discovered that CAD was an independent risk factor for cancer and vice versa. (2) Digestive, respiratory and urogenital cancers were independent risk factors for CAD. (3) We created a formula for the prediction of cancer among CAD patients. (4) ALT, usually considered a risk factor, was proven to be a protective factor for cancer in this study.

## Introduction

With the improvement of living standards, coronary atherosclerotic heart disease (CAD) and cancer have ranked as leading causes of death worldwide (1, 2). In 2017, a total of 126.5 million people were diagnosed with ischemic heart disease (IHD), and 10.6 million new cases of IHD were registered, resulting in 8.9 million deaths (1). Likewise, an estimated 19.3 million new cancer cases and almost 10.0 million cancer deaths were reported in 2020 (2). Considering the similar high morbidity and mortality of CAD and cancer, the relationship of their occurrence should be studied.

Coronary atherosclerotic heart disease and cancer were originally thought of as two separate diseases. Currently, accumulating studies have focused on the relevance of these two diseases. Cholesterol was reported to be positively associated with CAD and negatively associated with cancer, indicating a negative correlation between CAD and cancer (3), while some reports showed that colorectal cancer was positively related to CAD but with no significant difference (4). In our clinical work, we found that patients with cancer were more susceptible to CAD, and vice versa. From another point, CAD and cancer appear to share common risk factors. Male sex (5, 6), smoking (7, 8), diabetes (7, 9) and high body mass index (10, 11) have all been reported to be associated with the two diseases. All this evidence suggests a probable relationship between CAD and cancer. However, there is still a lack of large-scale studies on the relevance of CAD and cancer. Whether there are other factors involved in CAD and cancer, and whether each of these two diseases is a risk factor for the other needs further exploration.

In this retrospective study, applying single-factor and multivariate analysis methods, we analyzed the risk factors for CAD and cancer, trying to explore the answer to these questions.

## Materials and Methods

### Study Sample

In this retrospective study, we used data from 1,600 inpatients at TangDu Hospital from January 2012 to June 2019. The following patients were excluded from the present study: (1) patients who had not undergone coronary angiography (CAG); (2) patients with uncertain diagnoses of CAD and cancers; and (3) patients with incomplete medical data. The definition of CAD was stenosis of the coronary artery greater than or equal to 50% by CAG. The diagnosis of cancer was based on histopathological examination. This study was approved by the TangDu Hospital Medical Ethics Committee, which waived the requirement for informed consent (approved ID: K202106-07).

### Information Collection

We collected the patients’ basic information, medical histories and laboratory examination data, which were obtained from the Electronic Medical Records (EMRs) and Laboratory Information Management System (LIS) of TangDu Hospital. Hypertension was defined as systolic blood pressure (SBP) ≥ 140 mmHg and/or diastolic blood pressure (DBP) ≥ 90 mmHg or use of antihypertensive drugs. Diabetes was defined as fasting glucose level ≥ 7.0 mmol/L (126 mg/dl) or 2-h post load glucose level ≥ 11.0 mmol/L (200 mg/dl), or on the medications for diabetes currently. The laboratory examination included a routine blood examination, biochemical tests, coagulation tests and tumor biomarkers.

### Statistics

The statistical analysis was performed by SPSS version 26.0. A “*T* test” was applied for measurement data that conformed to a normal distribution, and for this test, the data are shown as the mean ± SD. Other measurement data that did not comply with a normal distribution were subjected to the Mann–Whitney test, for which the data are described as the median (interquartile range). The Pearson χ2 test was used for enumeration data, and the Mann–Whitney test was used for ranked data. These data are expressed as n (%). Multivariable analysis was conducted through binary logistic regression. Receiver operating characteristic (ROC) analysis and Youden’s index were performed to identify possible cutoff points and clinical sensitivities/specificities. The optimal coefficient of indicators was obtained by calculating the maximum value of the area under the curve (AUC) using MATLAB (version 7.0). A *p*-value of < 0.05 indicated statistical significance.

## Results

### Baseline Characteristics

#### Baseline Characteristics of Patients With and Without Coronary Atherosclerotic Heart Disease

A total of 1,600 patients, 996 with CAD and 604 without CAD, were enrolled in this study. Table 1 exhibits the comparisons of the baseline characteristics between patients with and without CAD. Through single-factor analysis, we concluded that the risk factors, including sex, age, smoking, drinking, and medical history of ischemic stroke, diabetes, and hypertension, were significantly correlated with CAD (all *p* < 0.01). Patients with CAD presented a higher index in glycaemia (*p* < 0.001), glycosylated hemoglobin (HbA1c) (*p* < 0.001), low-density lipoprotein-cholesterol (LDL-C) (*p* = 0.007), aspartate aminotransferase (AST) (*p* < 0.001), globulin (*p* = 0.043), direct bilirubin (*p* = 0.024), blood urea nitrogen (BUN) (*p* < 0.001), creatine (Cr) (*p* < 0.001), fibrinogen (FIB) (*p* < 0.001), and D-dimer (*p* < 0.001) than non-CAD patients. Compared with non-CAD patients, CAD patients had a lower level of red blood cell count (RBC) (*p* = 0.007), hemoglobin (HGB) (*p* = 0.018), high-density lipoprotein-cholesterol (HDL-C) (*p* < 0.001), apolipoprotein A (APOA) (*p* = 0.005), and albumin (*p* < 0.001) (Table 1).

**TABLE 1:** Baseline characteristics of patients with/without CAD and with/without cancer.

	Total	Non-CAD	CAD	*p*	Non-cancer	Cancer	*p*
Number	1,600	604	996		1,004	596	
**Basic information**							
Male gender (%)	1,062 (66.4)	318 (52.6)	744 (74.7)	< 0.001	638 (63.5)	424 (71.1)	0.002
Age (years)	60.7 ± 10.77	56.3 ± 10.47	63.4 ± 10.04	< 0.001	57.8 ± 10.88	65.7 ± 8.49	< 0.001
SBP (mmHg)	128.7 ± 16.56	126.9 ± 15.18	129.8 ± 17.26	0.001	128.2 ± 16.47	129.5 ± 16.69	0.141
DBP (mmHg)	78.0 ± 10.47	78.0 ± 10.00	78.1 ± 10.74	0.910	78.2 ± 10.68	77.8 ± 10.10	0.433
Smokers	714 (44.8)	207 (34.3)	507 (50.9)	< 0.001	413 (41.1)	301 (50.5)	< 0.001
Drinkers	290 (18.4)	52 (8.6)	238 (23.9)	< 0.001	162 (16.1)	128 (21.5)	0.008
**Medical histories**							
Hypertension	817 (51.1)	264 (43.7%)	553 (55.5%)	< 0.001	498 (49.6)	319 (53.7)	0.113
Diabetes	261 (16.3)	31 (5.1)	230 (23.1)	< 0.001	140 (13.9)	121 (20.3)	0.001
Ischemic stroke	119 (7.4)	29 (4.8)	90 (9.0)	0.002	69 (6.9)	50 (8.4)	0.264
CAD	996 (62.3)	–	–	–	526 (52.4)	470 (78.9)	< 0.001
**Routine blood examination**							
RBC (10^12^/L)	4.43 ± 1.15	4.53 ± 1.72	4.37 ± 0.56	0.007	4.50 ± 1.38	4.30 ± 0.55	0.001
HGB (g/L)	135.24 ± 17.58	136.55 ± 16.55	134.45 ± 18.14	0.018	137.75 ± 16.69	131.02 ± 18.24	< 0.001
PLT (10^9^/L)	200.01 ± 66.94	196.96 ± 63.13	201.84 ± 69.10	0.158	202.37 ± 66.77	196.04 ± 67.11	0.067
**Blood biochemical tests**							
Glycemia (mmol/L)	5.96 ± 1.84	5.55 ± 1.17	6.26 ± 2.15	< 0.001	5.95 ± 1.96	5.97 ± 1.59	0.881
HbA1c (%)	6.51 ± 1.57	5.83 ± 0.91	6.71 ± 1.67	< 0.001	6.36 ± 1.44	6.78 ± 1.74	0.020
TC (mmol/L)	3.90 ± 0.99	3.87 ± 0.91	3.92 ± 1.03	0.390	3.93 ± 0.92	3.83 ± 1.10	0.059
TG (mmol/L)	1.56 ± 1.04	1.54 ± 0.93	1.58 ± 1.10	0.489	1.61 ± 1.04	1.47 ± 1.02	0.018
HDL-C (mmol/L)	1.00 ± 0.25	1.04 ± 0.27	0.98 ± 0.24	< 0.001	1.01 ± 0.26	0.99 ± 0.24	0.093
LDL-C (mmol/L)	2.16 ± 0.76	2.09 ± 0.73	2.21 ± 0.78	0.007	2.19 ± 0.75	2.11 ± 0.78	0.061
APOA (mmol/L)	1.15 ± 0.25	1.20 ± 0.25	1.13 ± 0.25	0.005	1.12 ± 0.26	1.12 ± 0.23	0.062
APOB (mmol/L)	0.81 ± 0.26	0.81 ± 0.28	0.81 ± 0.26	0.985	0.82 ± 0.26	0.78 ± 0.26	0.107
AST (U/L)	25.00 (15.00)	24.00 (11.00)	26.00 (20.00)	< 0.001	26.00 (17.00)	24.00 (13.00)	0.001
ALT (U/L)	27.00 (20.00)	27.00 (19.00)	27.00 (21.00)	0.433	28.00 (21.00)	25.00 (19.00)	< 0.001
Albumin (g/L)	40.97 ± 4.07	41.86 ± 3.80	40.44 ± 4.14	< 0.001	41.43 ± 3.83	40.21 ± 4.35	< 0.001
Globulin (g/L)	26.35 ± 10.43	25.65 ± 6.21	26.76 ± 12.25	0.043	25.79 ± 6.56	27.27 ± 14.72	0.007
Total bilirubin (umol/L)	14.89 ± 9.32	15.48 ± 11.82	14.54 ± 7.42	0.055	14.97 ± 7.63	14.75 ± 11.59	0.651
Direct bilirubin (umol/L)	5.20 (3.20)	5.00 (3.00)	5.35 (3.24)	0.024	5.10 (3.17)	5.30 (3.31)	0.089
BUN (mmol/L)	5.33 ± 1.63	5.12 ± 1.51	5.46 ± 1.69	< 0.001	5.28 ± 1.60	5.42 ± 1.69	0.099
Cr (umol/L)	68.60 ± 27.49	64.83 ± 16.26	70.86 ± 32.22	< 0.001	67.86 ± 31.70	69.82 ± 18.41	0.169
**Coagulation tests**							
FIB (g/L)	2.83 ± 0.89	2.60 ± 0.70	2.97 ± 0.97	< 0.001	2.68 ± 0.80	3.08 ± 0.99	< 0.001
D-Dimer (ug/mL)	0.46 (0.51)	0.41 (0.46)	0.50 (0.56)	< 0.001	0.43 (0.47)	0.53 (0.61)	< 0.001
**Tumor markers**							
CA724 (U/mL)	1.70 (2.72)	1.49 (4.42)	1.77 (2.49)	0.552	1.21 (1.74)	1.80 (2.92)	0.063
CEA (ng/mL)	2.67 (2.84)	2.28 (1.79)	2.86 (3.60)	< 0.001	2.03 (1.14)	2.79 (3.25)	< 0.001
AFP (ng/mL)	2.46 (1.63)	2.63 (2.03)	2.34 (1.67)	0.014	2.48 (1.70)	2.45 (1.64)	0.970
Ferritin (ug/L)	158.00 (181.27)	187.00 (171.90)	149.50 (187.98)	0.072	190.80 (221.60)	154.60 (180.01)	0.179
NSE (ng/mL)	13.37 (7.07)	13.10 (7.64)	13.43 (6.94)	0.924	11.57 (3.77)	13.80 (7.37)	0.013
CA19-9 (U/mL)	11.88 (12.65)	9.72 (9.46)	12.90 (12.92)	0.030	8.54 (7.62)	12.73 (12.98)	0.010
CA125 (U/mL)	13.44 (12.71)	12.17 (10.93)	13.75 (12.91)	0.236	9.91 (7.13)	13.92 (13.31)	< 0.001
CA153 (U/mL)	11.99 (8.62)	11.24 (4.88)	12.43 (9.91)	0.227	11.53 (10.81)	12.02 (7.77)	0.837
CYFRA21-1 (ng/mL)	3.05 (2.99)	2.55 (2.43)	3.14 (3.70)	0.008	2.42 (1.89)	3.14 (3.59)	0.007
CA50 (U/mL)	8.03 (6.44)	8.03 (6.76)	8.02 (6.58)	0.733	7.39 (3.16)	8.04 (6.72)	0.439
SCC (ng/mL)	0.48 (0.24)	0.46 (0.24)	0.50 (0.24)	0.858	0.51 (0.10)	0.48 (0.24)	0.491
Cancer	596 (37.3)	126 (20.9)	470 (47.2)	< 0.001	–	–	–
**Cancer types**				< 0.001			
Respiratory system^a^	230 (14.4)	46 (7.6)	184 (18.5)				
Digestive system^b^	220 (13.8)	46 (7.6)	174 (17.5)				
Urogenital system^c^	84 (5.3)	12 (2.0)	72 (7.2)				
Superficial organ^d^	62 (3.9)	22 (3.6)	40 (4.0)				

*^a^Respiratory system cancer includes nasopharyngeal and pulmonary cancers.*

*^b^Digestive system cancer includes esophageal, gastric, colon, liver, gallbladder and pancreatic cancers.*

*^c^Urogenital system cancer includes uterine, ovarian, renal, ureteral, bladder, prostatic and penile cancers.*

*^d^Superficial organ cancer includes thyroid, breast and other superficial organ cancers.*

*ALT, alanine aminotransferase; AFP, alpha fetoprotein; APOA/APOB, apolipoprotein A/B; AST, aspartate aminotransferase; BUN, blood urea nitrogen; CAD, coronary atherosclerotic disease; CEA, carcinoembryonic antigen; Cr, creatine; CYFRA21-1, cytokeratin 19 fragment; DBP/SBP, diastolic/systolic blood pressure; FIB, fibrinogen; HbA1c, glycosylated hemoglobin; HDL/LDL-C, high/low-density lipoprotein-cholesterol; HGB, hemoglobin; NSE, neuron specific enolase; PLT, platelet; SCC, squamous cell carcinoma antigen; TC, total cholesterol and TG, triglyceride.*

*Measurement values that conform to normal distribution are described as mean ± SD, others as median (interquartile range). Enumeration data was expressed as n (%).*

As shown in Table 1, a challenging finding was that of all the patients we monitored in this study, 47.2% of the CAD patients had a history of cancer, and for non-CAD patients, this figure was 20.9% (*p* < 0.001). Furthermore, there was a significant difference in the distribution of cancer types between the non-CAD and CAD groups (*p* < 0.001). CAD patients had the highest prevalence of respiratory cancer (18.5%), followed by digestive (17.5%), urogenital (7.2%), and superficial organ (4.0%) cancers. Patients without CAD had the highest prevalence of respiratory and digestive cancer (both 7.6%), followed by superficial organ (3.6%), and urogenital (2.0%) cancer. Our analysis also proved that patients with CAD had a higher level of carcinoembryonic antigen (CEA) (*p* < 0.001), CA19-9 (*p* = 0.030) and cytokeratin 19 fragment (CYFRA21-1) (*p* = 0.008) than non-CAD patients (Table 1).

#### Baseline Characteristics of Patients With and Without Cancer

The study subjects included 596 cancer patients and 1,004 non-cancer patients. Table 1 exhibits the comparisons of the baseline characteristics between patients with and without cancer. The single-factor analysis revealed that factors, including sex, age, smoking, drinking and medical history of diabetes, were significantly different (*p* < 0.01) between the two groups. Patients with cancer showed a higher index in HbA1c (*p* = 0.020), globulin (*p* = 0.007), FIB (*p* < 0.001), D-dimer (*p* < 0.001), CEA (*p* < 0.001), neuron specific enolase (NSE) (*p* = 0.013), CA19-9 (*p* = 0.010), CA125 (*p* < 0.001), CYFRA21-1 (*p* = 0.007), but a lower level of RBC (*p* = 0.001), HGB (*p* < 0.001), triglyceride (TG) (*p* = 0.018), AST (*p* = 0.001), alanine aminotransferase (ALT) (*p* < 0.001) and albumin (*p* < 0.001) than patients without cancer. Surprisingly, of all the samples we analyzed, 78.9% of the cancer patients had a history of CAD, and for non-cancer patients, this figure was 52.4% (*p* < 0.001) (Table 1).

#### Baseline Characteristics of Patients With or Without Coronary Atherosclerotic Heart Disease/Cancer

Table 2 summarizes the comparisons of the baseline characteristics of patients in CAD-/cancer- (*n* = 478), CAD-/cancer + (*n* = 126), CAD + /cancer- (*n* = 526) and CAD + /cancer + groups (*n* = 470). The CAD + /cancer + group exhibited an older age than the CAD-/cancer- (66.6 ± 8.41 vs. 54.7 ± 10.47), CAD-/cancer + (66.6 ± 8.41 vs. 62.5 ± 8.02) and CAD + /cancer- (66.6 ± 8.41 vs. 60.6 ± 10.52) groups (all *p* < 0.001). Lower levels of albumin and higher levels of FIB and D-dimer were shown in the CAD + /cancer + group than the CAD-/ cancer-, CAD-/cancer + and CAD + /cancer- groups (all *p* < 0.05).

**TABLE 2:** Baseline characteristics of patients in CAD–/ cancer–, CAD-/cancer + , CAD + /cancer– and CAD + /cancer + groups.

	CAD-/cancer-	CAD-/cancer +	CAD + /cancer-	CAD + /cancer +	p1*	p2^#^	p3^†^
Number	478	126	526	470			
**Basic information**							
Male gender (%)	251 (52.5)	67 (53.2)	387 (73.6)	357 (76.0)	< 0.001	<0.001	0.388
Age (years)	54.7 ± 10.47	62.5 ± 8.02	60.6 ± 10.52	66.6 ± 8.41	< 0.001	<0.001	< 0.001
SBP (mmHg)	127.0 ± 15.35	126.8 ± 14.58	129.4 ± 17.36	130.2 ± 17.15	0.002	0.039	0.445
DBP (mmHg)	78.1 ± 10.02	77.6 ± 9.93	78.3 ± 11.25	77.8 ± 10.15	0.661	0.864	0.483
Smokers	158 (33.1)	49 (38.9)	255 (48.5)	252 (53.6)	< 0.001	0.003	0.168
Drinkers	35 (7.3)	17 (13.5)	127 (24.1)	111 (23.6)	< 0.001	0.011	0.793
**Medical histories**							
Hypertension	211 (44.1)	53 (42.1)	287 (54.6)	266 (56.6)	< 0.001	0.003	0.471
Diabetes	28 (5.9)	3 (2.4)	112 (21.3)	118 (25.1)	< 0.001	<0.001	0.154
Ischemic stroke	14 (2.9)	15 (11.9)	55 (10.5)	35 (7.4)	0.002	0.109	0.098
Routine blood examination							
RBC (10^12^/L)	4.57 ± 1.92	4.38 ± 0.47	4.44 ± 0.55	4.28 ± 0.57	0.002	0.050	< 0.001
HGB (g/L)	137.27 ± 16.33	133.83 ± 17.12	138.19 ± 17.00	130.27 ± 18.47	< 0.001	0.051	< 0.001
PLT (10^9^/L)	200.22 ± 61.83	184.67 ± 66.65	204.31 ± 70.93	199.08 ± 66.97	0.786	0.032	0.233
**Blood biochemical tests**							
Glycemia (mmol/L)	5.57 ± 1.23	5.46 ± 0.93	6.37 ± 2.46	6.13 ± 1.72	< 0.001	<0.001	0.114
HbA1c (%)	5.83 ± 0.91	5.85 ± 0.96	6.56 ± 1.56	6.91 ± 1.79	< 0.001	0.001	0.085
TC (mmol/L)	3.91 ± 0.90	3.72 ± 0.94	3.96 ± 0.95	3.86 ± 1.14	0.518	0.263	0.149
TG (mmol/L)	1.59 ± 0.98	1.32 ± 0.60	1.63 ± 1.09	1.51 ± 1.11	0.300	0.109	0.118
HDL-C (mmol/L)	1.05 ± 0.28	1.02 ± 0.23	0.98 ± 0.25	0.98 ± 0.24	< 0.001	0.120	0.930
LDL-C (mmol/L)	2.11 ± 0.73	2.01 ± 0.74	2.26 ± 0.77	2.14 ± 0.79	0.674	0.151	0.024
APOA (mmol/L)	1.20 ± 0.26	1.19 ± 0.22	1.14 ± 0.26	1.11 ± 0.23	0.003	0.077	0.200
APOB (mmol/L)	0.82 ± 0.28	0.76 ± 0.25	0.82 ± 0.25	0.79 ± 0.27	0.339	0.576	0.185
AST (U/L)	24 (11)	25 (12)	27 (25)	23 (14)	0.947	0.365	< 0.001
ALT (U/L)	27 (21)	26 (21)	29 (23)	24 (18)	0.003	0.121	< 0.001
Albumin (g/L)	42.01 ± 3.70	41.30 ± 4.13	40.91 ± 3.86	39.92 ± 4.37	< 0.001	0.002	< 0.001
Globulin (g/L)	25.42 ± 6.22	26.51 ± 6.11	26.12 ± 6.84	27.47 ± 16.27	0.012	0.523	0.087
Total bilirubin (umol/L)	14.87 ± 7.32	14.06 (8.45)	13.39 (7.82)	12.60 (7.86)	0.050	0.029	0.037
Direct bilirubin (umol/L)	5.35 ± 3.23	5.40 (3.64)	5.40 (3.22)	5.30 (3.30)	0.126	0.617	0.982
BUN (mmol/L)	5.06 ± 1.42	5.33 ± 1.78	5.47 ± 1.72	5.44 ± 1.67	< 0.001	0.536	0.753
Cr (umol/L)	64.38 ± 16.43	66.54 ± 15.55	70.99 ± 40.59	70.71 ± 19.02	< 0.001	0.025	0.889
**Coagulation tests**							
FIB (g/L)	2.52 ± 0.65	2.89 ± 0.80	2.83 ± 0.89	3.13 ± 1.02	< 0.001	0.013	< 0.001
D-Dimer (ug/mL)	0.40 (0.44)	0.44 (0.48)	0.47 (0.51)	0.55 (0.64)	< 0.001	0.001	< 0.001

*p1 represents comparison between the CAD-/cancer- and CAD + /cancer + groups.

*^#^p2 represents comparison between the CAD-/cancer + and CAD + /cancer + groups.*

*^†^p3 represents comparison between the CAD + /cancer- and CAD + /cancer + groups.*

*ALT, alanine aminotransferase; AFP, alpha fetoprotein; APOA/APOB, apolipoprotein A/B; AST, aspartate aminotransferase; BUN, blood urea nitrogen; CAD, coronary atherosclerotic disease; Cr, creatine; DBP/SBP, diastolic/systolic blood pressure; FIB, fibrinogen; HbA1c, glycosylated hemoglobin; HDL/LDL-C, high/low-density lipoprotein-cholesterol; HGB, hemoglobin; PLT, platelet; TC, total cholesterol and TG, triglyceride.*

*Measurement values that conform to normal distribution are described as mean ± SD, others as median (interquartile range). Enumeration data was expressed as n (%).*

### Multivariate Analysis for Risk Factors for Coronary Atherosclerotic Heart Disease

In this study, multivariate logistic regression analysis was performed to assess factors related to CAD, with CAD as the dependent variable and factors including age, sex, drinking, smoking, HGB, serum lipids (total cholesterol (TC), TG, HDL-C, LDL-C), liver function indexes (AST, ALT, direct bilirubin, albumin and globulin), Cr, BUN, D-dimer, fibrinogen, and history of hypertension, diabetes, stroke and cancers as the independent variables. The data obtained in the process of multivariate analyses proved a strong association between CAD and the following characteristics: male (OR: 2.558, 95% CI: 1.812–3.610, *p* < 0.001), age ≥ 45 years (45–60 years: OR: 2.010; 60–75 years: OR: 3.834; ≥ 75 years: OR: 9.791; all *p* < 0.05), hypertension (OR: 1.464, 95% CI: 1.114–1.924, *p* = 0.006), diabetes (OR: 4.879, 95% CI: 3.052–7.800, *p* < 0.001), drinking (OR: 3.333, 95% CI: 2.186–5.082, *p* < 0.001), an LDL-C level ≥ 1.8 mmol/L (1.8–2.6 mmol/L: OR: 1.382; ≥ 2.6 mmol/L: OR: 1.641; all *p* < 0.05), an AST level ≥ 40 U/L (OR: 3.053, 95% CI: 1.976–4.716, *p* < 0.001), an FIB level ≥ 3.5 g/L (OR: 2.599, 95% CI: 1.631–4.143, *p* < 0.001), and an HGB level ≥ 131 g/L (OR: 0.636, 95% CI: 0.455–0.889, *p* = 0.008) (Table 3).

**TABLE 3:** Logistic regression analysis for influencing factors for CAD.

	OR	95% CI	*p*
Gender: Female	1		
Male	2.558	1.812–3.610	< 0.001
Age (years) < 45	1		
45–60	2.010	1.211–3.336	0.007
60–75	3.834	2.257–6.515	< 0.001
>75	9.791	4.524–21.192	< 0.001
Hypertension	1.464	1.114–1.924	0.006
Diabetes	4.879	3.052–7.800	< 0.001
Cancer	2.024	1.475–2.778	< 0.001
Cancer types			
No cancer	1		
Respiratory system ^a^	1.981	1.236–3.175	0.005
Digestive system ^b^	1.899	1.177–3.064	0.009
Urogenital system ^c^	3.595	1.696–7.620	0.001
Superficial organ ^d^	1.407	0.696–2.843	0.342
Drinkers	3.333	2.186–5.082	< 0.001
HGB (g/L) ≥ 131	0.636	0.455–0.889	0.008
LDL-C (mmol/L) < 1.8	1		
1.8–2.6	1.382	1.005–1.902	0.047
≥2.6	1.641	1.148–2.345	0.007
AST (U/L) < 20	1		
20–40	0.999	0.734–1.358	0.993
≥40	3.053	1.976–4.716	< 0.001
FIB (g/L) < 2.5	1		
2.5–3.5	1.301	0.972–1.742	0.077
≥3.5	2.599	1.631–4.143	< 0.001

*^a^Respiratory system cancer includes nasopharyngeal and pulmonary cancers.*

*^b^Digestive system cancer includes esophageal, gastric, colon, liver, gallbladder and pancreatic cancers.*

*^c^Urogenital system cancer includes uterine, ovarian, renal, ureteral, bladder, prostatic and penile cancers.*

*^d^Superficial organ cancer includes thyroid, breast and other superficial organ cancers.*

*AST, aspartate aminotransferase; BP, blood pressure; CAD, coronary atherosclerotic disease; FIB, fibrinogen and HGB, hemoglobin.*

Importantly, an interesting finding was that patients with cancer were particularly vulnerable to CAD (OR: 2.024, 95% CI: 1.475–2.778, *p* < 0.001). Respiratory, digestive and urogenital cancers were significantly associated with a higher risk of CAD compared with no cancer (OR: 1.981, 95% CI: 1.236–3.175, *p* = 0.005; OR: 1.899, 95% CI: 1.177–3.064, *p* = 0.009; OR: 3.595, 95% CI: 1.696–7.620, *p* = 0.001), after adjustment for age, sex, drinking, smoking, history of hypertension, diabetes and stroke and HGB, TC, TG, HDL-C, LDL-C, AST, ALT, direct bilirubin, albumin, globulin, Cr, BUN, D-dimer and FIB levels. The multivariate logistic regression results are visualized in Table 3.

### Multivariate Analysis for Risk Factors for Cancer

Then, a multivariate logistic regression method was used to analyze the factors related to cancer, with cancer as the dependent variable and factors including age, sex, drinking, smoking, HGB, serum lipids (TC, TG, HDL-C, LDL-C), liver function indexes (AST, ALT, albumin, globulin), D-dimer, FIB, and history of diabetes and CAD as the independent variables. Through the multivariate analyses, we found that age (45–60 years: OR: 5.359; 60–75 years: OR: 15.193; ≥ 75 years: OR = 18.179; all *p* ≤ 0.001), smoking (OR: 1.651, 95% CI: 1.247–2.184, *p* < 0.001), an FIB levels (2.5–3.5 g/L: OR: 1.354; ≥ 3.5 g/L: OR: 1.926; all *p* < 0.05) were positively correlated with cancer, while HGB levels (OR: 0.743, 95% CI: 0.560–0.986, *p* = 0.040) was negatively related to cancer. There was also a significant difference in the incidence of cancer between the ALT level ≥ 40 U/L group and the ALT level < 20 U/L group (OR: 0.490, 95% CI: 0.333–0.722, *p* < 0.001). And it is worth noticing that CAD patients were more prone to suffer from cancer compared to those without CAD (OR: 2.157, 95% CI: 1.603–2.902, *p* < 0.001) (Table 4).

**TABLE 4:** Logistic regression analysis for influencing factors for cancer.

	OR	95% CI	*p*
Age (years) < 45	1		
45–60	5.359	1.908–15.048	0.001
60–75	15.193	5.434–42.478	< 0.001
≥75	18.179	6.053–54.599	< 0.001
CAD	2.157	1.603–2.902	< 0.001
Smokers	1.651	1.247–2.184	< 0.001
HGB (g/L) ≥ 131	0.743	0.560–0.986	0.040
ALT (U/L) < 20	1		
20–40	0.849	0.630–1.143	0.279
≥40	0.490	0.333–0.722	< 0.001
FIB (g/L) < 2.5	1		
2.5–3.5	1.354	1.013–1.809	0.041
≥3.5	1.926	1.315–2.821	0.001

*ALT, alanine aminotransferase; CAD, coronary atherosclerotic disease; FIB, fibrinogen and HGB, hemoglobin.*

### Multivariate Analysis for Risk Factors for Combined Coronary Atherosclerotic Heart Disease and Cancer

The risk factors for combined CAD and cancer were determined by multivariate logistic regression, as shown in Table 5. The CAD + /cancer + group was compared with the CAD-/ cancer-, CAD-/cancer + and CAD + /cancer- groups, respectively. After adjustment for age, sex, history of hypertension, diabetes and ischemic stroke, drinking, smoking, serum lipids (TC, TG, HDL-C, LDL-C), liver function indexes (AST, ALT, albumin, globulin), Cr, BUN and D-dimer, an FIB level ≥ 2.5 g/L was positively associated with combined CAD and cancer, while an HGB level ≥ 131 g/L was significantly associated with a low risk of combined CAD and cancer.

**TABLE 5:** Logistic regression analysis for influencing factors for combined CAD and cancer.

	Model 1*		Model 2^#^		Model 3^†^	
			
	OR (95% CI)	*p*	OR (95% CI)	*p*	OR (95% CI)	*p*
Age (years) < 45	1		1		1	
45–60	8.164 (2.203–30.252)	0.002	0.589 (0.057–6.049)	0.656	3.626 (1.061–12.389)	0.040
60–75	41.179 (11.011–153.998)	< 0.001	0.598 (0.060–5.996)	0.662	8.256 (2.437–27.967)	0.001
>75	90.253 (20.792–391.762)	< 0.001	4.862 (0.324–72.907)	0.252	12.362 (3.399–44.964)	< 0.001
HGB (g/L) ≥ 131	0.475 (0.298–0.758)	0.002	0.414 (0.231–0.741)	0.003	0.618 (0.441–0.865)	0.005
FIB (g/L) < 2.5	1		1		1	
2.5–3.5	1.751 (1.135–2.702)	0.011	1.904 (1.071–3.385)	0.028	1.508 (1.058–2.150)	0.023
≥3.5	5.054 (2.651–9.634)	< 0.001	3.025 (1.380–6.631)	0.006	2.038 (1.317–3.152)	0.001

*Model 1 represents comparison between the CAD-/cancer- and CAD + /cancer + groups.

*#Model 2 represents comparison between the CAD-/cancer + and CAD + /cancer + groups.*

*†Model 3 represents comparison between the CAD + /cancer- and CAD + /cancer + groups.*

*CAD, coronary atherosclerotic disease; FIB, fibrinogen and HGB, hemoglobin.*

## The Formula Prediction for Cancer

Our data showed that CAD was closely related to cancer. We explored a way to monitor the occurrence of cancer among CAD patients. Having confirmed that age and HGB, ALT and FIB levels were independent risk factors for cancer, we tried to give them weighting coefficients and generate a dependent variable Y through MATLAB software (version 7.0):

Y = 0.205 × 10^–1^ age − 0.595 × 10^–2^ HGB − 0.116 × 10^–1^ ALT + 0.135 FIB

**FIGURE 1:**
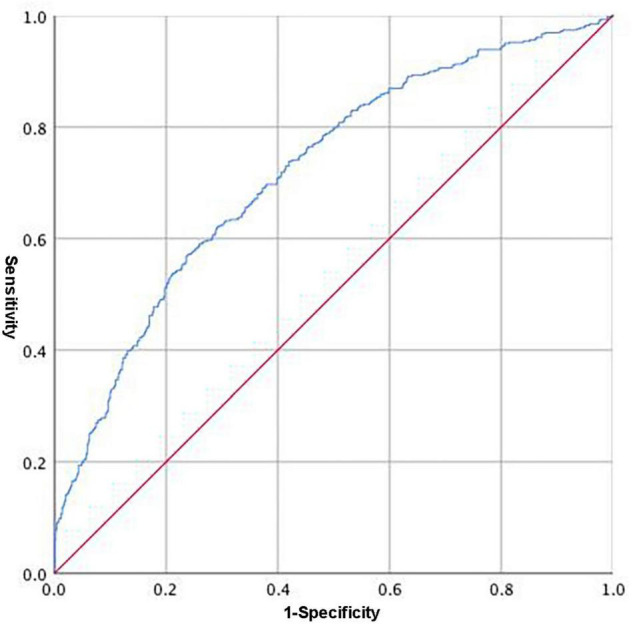
Receiver operating characteristic (ROC) of the integrated variable (Y = 0.205 × 10^–1^ age − 0.595 × 10^–2^ HGB − 0.116 × 10^–1^ ALT + 0.135 FIB) predicting the occurrence of cancer among CAD patients.

We calculated Youden’s index to identify the optimal cutoff point, which was 0.6044, with a clinical sensitivity/specificity of 0.617/0.711, respectively. The maximum area under the curve (AUC) was 0.720 (95% CI, 0.688–0.752, Figure 1).

## Discussion

Coronary atherosclerotic heart disease and cancer share similar epidemiological characteristics. And in our clinical work, we found a very interesting phenomenon in which patients who had been diagnosed with cancer were more susceptible to CAD. Therefore, we tried to determine whether there is any relationship between CAD and cancer. We reviewed the raw data of 1,600 patients with/without CAD and cancer in Tangdu Hospital, which led us to the following findings. (1) We discovered that CAD was an independent risk factor for cancer and vice versa. (2) Digestive, respiratory and urogenital cancers were independent risk factors for CAD. (3) We created a formula for the prediction of cancer among CAD patients. (4) ALT, usually considered a risk factor, was proven to be a protective factor for cancer in this study.

To the best of our knowledge, this was the first study to verify that CAD and cancer are independent risk factors for each other. CAD and cancer were originally thought of as two separate diseases. Currently, accumulating studies have focused on the relevance of these two diseases. In the 1980s and 1990s, Smith GD and Kagan A reported that the mortality and morbidity of CAD increased with increasing cholesterol concentration, while the mortality and morbidity of cancer showed an inverse trend with cholesterol levels (3, 12). These early epidemiological studies may indicate a negative correlation between the two diseases. With the study of the relationship between them, this view has changed greatly. The most recent studies showed that the risk of recurrence and cancer-specific death in patients with early cardiovascular events after cancer diagnosis has increased. In addition, a number of studies have shown that aspirin and statins, the drugs that benefit CAD patients, also have protective effects on patients with cancer (13–15). All these studies may suggest a possible connection between cancer and CAD. Our study creatively pointed out that CAD and cancer are independent risk factors for each other. Then, we explored the distribution of cancer in patients with or without CAD to determine which specific kind of cancer makes patients more susceptible to CAD. Several studies have involved this question, but have not offered a firm conclusion for that. In 2006 and 2007, Chan et al. delivered two publications reporting that patients with colorectal neoplasms suffered a higher prevalence of CAD than patients without colorectal neoplasms, while colorectal cancer was not significantly associated with CAD by multivariate logistic regression (4, 16). In 2021, a retrospective study showed that lung, colorectal, gastric, breast, and thyroid cancer patients suffered from a relatively high prevalence of CVD, but this study did not include non-cancer patients (17). In our study, respiratory system, digestive system and urogenital system cancers were significantly associated with a higher risk of CAD compared with no cancer, with ORs of 1.981, 1.899 and 3.595, respectively (all *p* < 0.01), providing evidence for enhancing the prevention and screening of CAD in these patients.

Coronary atherosclerotic heart disease is mostly attributed to the occurrence of cardiovascular risk factors (e.g., hypertension, diabetes, smoking, age, hypercholesterinemia, and adiposity) (18). These are not solely risk factors for cardiovascular diseases, but also increase the risk of cancer (19). Our study applied single-factor and multivariate logistic analyses and found that CAD, cancer and combined CAD and cancer shared a common risk factor, FIB levels (20, 21), and a common protective factor, HGB levels (22, 23). The phenomenon that CAD and cancer possess various similarities and interactions suggests a shared biology for the two diseases. Inflammation appears to be a major unifying factor in the etiology and progression of these diseases (24, 25). Common conditions such as a high level of FIB induce inflammation (26), which may, in part, explain why CVD and cancer share several similarities. Except for the common influencing factors, we also concluded that sex, age (7), diabetes (27), drinking (28) and AST levels (29) were risk factors for CAD, and age (30) and smoking (8) were risk factors for cancer, which is similar to the findings of previous studies. ALT levels were found to be significantly higher in patients with cancer (31), and elevated levels of ALT were reported to be correlated with death in cancer patients (32). This study showed an opposite result, with the conclusion that ALT is a protective factor for cancer. However, the underlying mechanism is unclear. We need larger-scale studies to further clarify this.

Cancer is usually diagnosed at an advanced stage, leading to a poor prognosis. The reason is that there are no specific symptoms or effective diagnostic means in the early stage of cancer. Patients with CAD were more susceptible to cancer than non-CAD patients. Therefore, we attempted to predict the probability of cancer in CAD patients. The traditional method of most cancer diagnoses is tissue biopsy or imaging examination (33, 34). However, these are invasive, demanding, and time-consuming. A biomarker for cancer prediction would have tremendous clinical benefits in reducing the rate of invasive procedures, the time to diagnosis and costs. Therefore, study intended to use the most common items of blood examinations to address these problems. We gave a specific weighting coefficient to independent influencing factors of cancer(including age and HGB, ALT and FIB levels) and obtained an integrated parameter named Y, with a clinical sensitivity/specificity of 0.617/0.711, respectively: Y = 0.205 × 10^–1^ age − 0.595 × 10^–2^ HGB − 0.116 × 10^–1^ ALT + 0.135 FIB. The study proposed a new non-invasive method for the prediction of cancer among CAD patients. We still need more data to demonstrate the accuracy and effectiveness of this formula. This is the direction of our future clinical work and research.

### Limitations

The present study has several limitations. First, as a retrospective study, some laboratory examinations, such as the tumor biomarkers of the patients, were incomplete. Further cohort studies are needed to verify the conclusions of this study. Second, this was a descriptive study of patients admitted to a single center; therefore, this study is not representative of the entire population. Finally, the sample size was relatively small. Subsequent studies are needed to provide additional explanations.

## Conclusion

(1) We discovered that CAD was an independent risk factor for cancer and vice versa. (2) Digestive, respiratory and urogenital cancers were independent risk factors for CAD. (3) We created a formula for the prediction of cancer among CAD patients. (4) ALT, usually considered a risk factor, was proven to be a protective factor for cancer in this study.

## Data Availability Statement

The raw data supporting the conclusions of this article will be made available by the authors, without undue reservation.

## Ethics Statement

The studies involving human participants were reviewed and approved by Ethics Committee of Tangdu Hospital of the Fourth Military Medical University. Written informed consent for participation was not required for this study in accordance with the national legislation and the institutional requirements.

## Author Contributions

JJL, XL, and LS designed the study and drafted the manuscript. MC, XW, JL, and XY extracted and verified the data. JZ, YL, RC, and LS analyzed the data. JZ, YC, and PL revised the manuscript. JJL and XL incorporated comments from the co-authors and finalized the manuscript. All authors approved the final version of the paper.

## Conflict of Interest

The authors declare that the research was conducted in the absence of any commercial or financial relationships that could be construed as a potential conflict of interest.

## Publisher’s Note

All claims expressed in this article are solely those of the authors and do not necessarily represent those of their affiliated organizations, or those of the publisher, the editors and the reviewers. Any product that may be evaluated in this article, or claim that may be made by its manufacturer, is not guaranteed or endorsed by the publisher.
